# Supplementary material for: Computational modeling-directed combination treatment with etanercept and mifepristone mitigates neuroinflammation in a mouse model of Gulf War Illness
Source: PLoS One. 2026 Mar 17;21(3):e0324577. doi: 10.1371/journal.pone.0324577 (PMC12994794; doi:10.1371/journal.pone.0324577)
Supplement: S1 Appendix — (DOCX) [file pone.0324577.s001.docx]

**S1 Appendix. Etanercept and mifepristone dose response figure and methods.**

**
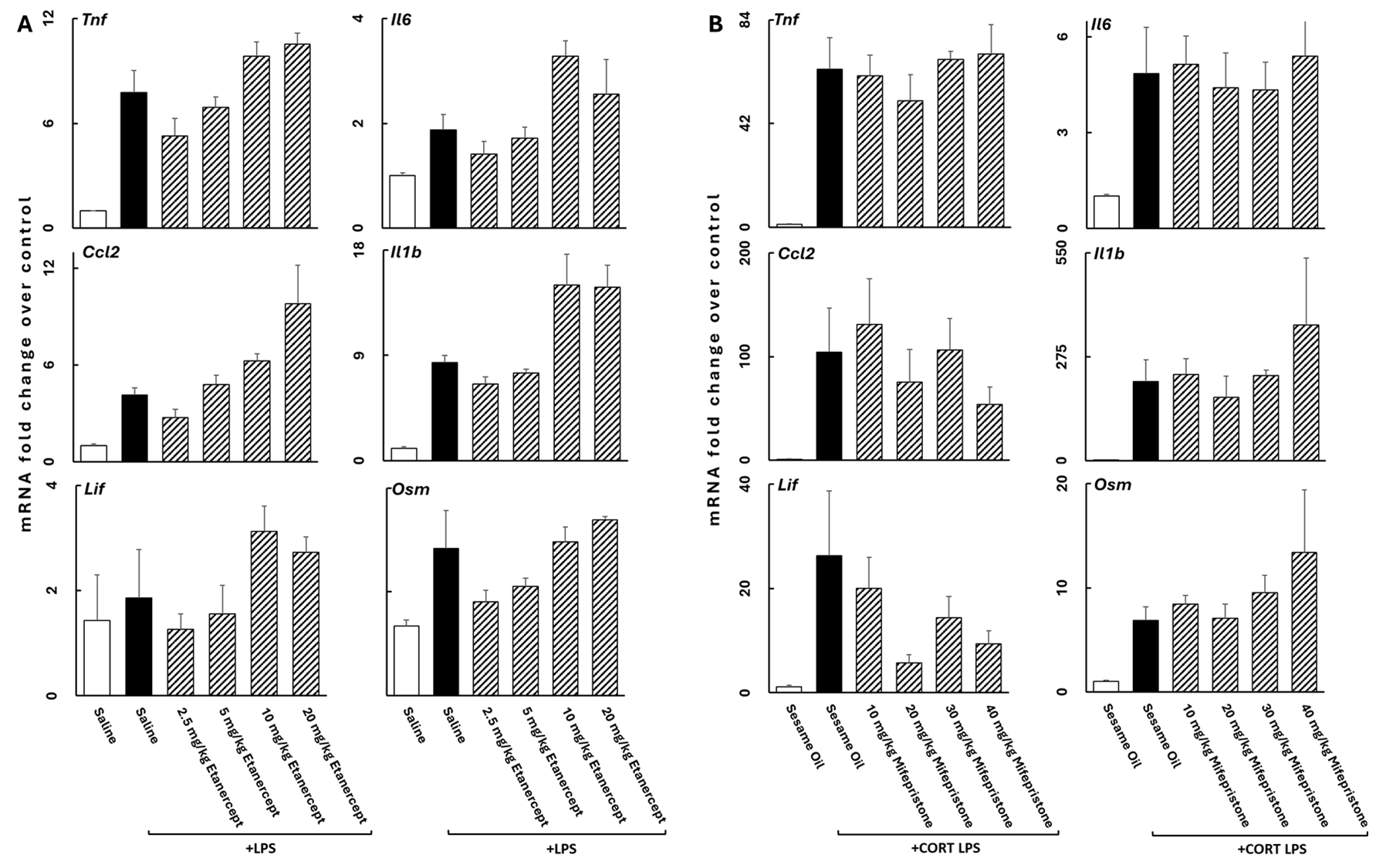
**

**Fig 1. Dose response evaluation for etanercept and mifepristone. A)** Mice (*N*=3-5 mice/group) were given etanercept (2.5, 5, 10, or 20 mg/kg, s.c.) 24 hours before receiving a single dose of lipopolysaccharide (LPS; 2.0 mg/kg, s.c.). Cortex was collected at 2 hours post-LPS exposure for qPCR analysis to evaluate changes in the mRNA expression of several inflammatory cytokines. **B)** Mice (*N*=3-5 mice/group) were given corticosterone (CORT; 200 mg/L in 0.6% ethanol) in the drinking water for seven days followed by a single injection of LPS (2 mg/kg, s.c.). Mifepristone (10, 20, 30, or 40 mg/kg, i.p. in sesame oil) was given on Day 4. Cortex was collected at 6 hours post-LPS exposure for qPCR analysis to evaluate changes in the mRNA expression of several inflammatory cytokines.

**Methods**

**Animals and exposure paradigms**

All animal procedures were performed within protocols approved by the US Centers for Disease Control and Prevention-Morgantown Institutional Animal Care and Use Committee (13-JO-M-021) and the US Army Medical Research and Development Command Animal Care and Use Review Office (GW120045.01) in an AAALAC International accredited facility. Adult male C57BL/6J mice (6-8 weeks old, ~30g; The Jackson Laboratory, Bar Harbor, ME, USA; RRID:IMSR_JAX:000664) were single housed and allowed to acclimate for at least one week prior to the exposures. Mice were given food (Harlan 7913 irradiated NIH-31 modified 6% rodent chow) and water ad libitum and received daily health checks from animal husbandry personnel.

**Etanercept dose response**

Mice (N=24) were given etanercept (2.5, 5, 10, or 20 mg/kg, s.c.) 24 hours before receiving a single dose of lipopolysaccharide (LPS; 2.0 mg/kg, s.c.). This higher dose of LPS was chosen to elicit a prototypical LPS-induced neuroinflammatory response. The mice were sacrificed by decapitation at 2 hrs post-LPS, the peak of the LPS-induced neuroinflammatory response [1]. Prior to dosing, mice were randomly assigned to one of 6 treatment groups: Saline (N=3), LPS (N=3), 2.5 mg/kg Etanercept + LPS (N=4), 5 mg/kg Etanercept + LPS (N=5), 10 mg/kg Etanercept + LPS (N=5), and 20 mg/kg Etanercept + LPS (N=4).

**Mifepristone dose response**

Mice (N=29) were given corticosterone (CORT; 200 mg/L in 0.6% ethanol) in the drinking water for seven days followed by a single injection of LPS (2 mg/kg, s.c.). Mifepristone (10, 20, 30, or 40 mg/kg, i.p. in NF grade sesame oil) was given on Day 4. The mice were sacrificed by decapitation at 6hrs post-LPS, the peak of the CORT-modified LPS-induced neuroinflammatory response [1]. Prior to dosing, mice were randomly assigned to one of 6 treatment groups: Sesame oil vehicle (N=5), CORT LPS (N=4), 10 mg/kg Mifepristone + CORT LPS (N=5), 20 mg/kg Mifepristone + CORT LPS (N=5), 30 mg/kg Mifepristone + CORT LPS (N=5), and 40 mg/kg Mifepristone + CORT LPS (N=4).

**Brain dissection, tissue preparation, and real-time qPCR**

Whole brains were dissected into target brain areas as described in the manuscript. Frontal cortex samples were used for real-time qPCR evaluation of inflammatory cytokines as described in the manuscript.

**Data Availability**

All data are available in Mendeley Data (Kelly, Kimberly; Felton, Christopher; Billig, Brenda; Yilmaz, Ali; O'Callaghan, James; Michalovicz, Lindsay (2026), “qRT-PCR of Inflammatory Mediators in Brain of Etanercept and mifepristone treatment in Gulf War Illness model”, Mendeley Data, V1, doi: 10.17632/pxtr6s225y.1).

**References**

1. Kelly, K. A., Michalovicz, L. T., Miller, J. V., Castranova, V., Miller, D. B., & O'Callaghan, J. P. Prior exposure to corticosterone markedly enhances and prolongs the neuroinflammatory response to systemic challenge with LPS. PloS one, 2018;13(1): e0190546.
